# Short-Term Associations Between Fat-Free Mass Preservation and Glycaemic Markers During Tirzepatide Therapy: A Secondary Exploratory Analysis

**DOI:** 10.3390/nu18071092

**Published:** 2026-03-29

**Authors:** Luigi Schiavo, Biagio Santella, Monica Mingo, Gianluca Rossetti, Marcello Orio, Luigi Cobellis, Francesco Cobellis, Vincenzo Pilone

**Affiliations:** 1 Department of Medicine, Surgery and Dentistry “Scuola Medica Salernitana”, University of Salerno, 84081 Baronissi, Italy; bsantella@unisa.it (B.S.); mmingo@unisa.it (M.M.); 2 General and Bariatric Surgery Unit, Abano Terme Policlinic, 35031 Padova, Italy; gianlucarossetti@yahoo.it; 3 Medical and Diabetological Center CMSO, 84123 Salerno, Italy; marcello.orio@gmail.com; 4 Unit of General Surgery, Casa Di Cura “Prof. Dott. Luigi Cobellis”, 84078 Vallo Della Lucania, Italycobellisfc@gmail.com (F.C.); 5 Public Health Department, University of Naples Federico II, 80131 Naples, Italy; vincenzo.pilone@unina.it

**Keywords:** tirzepatide, obesity, fat-free mass, body composition, glycaemic markers, insulin resistance, resting metabolic rate, exploratory analysis

## Abstract

Background/Objectives: Tirzepatide (TZP), a dual glucose-dependent insulinotropic polypeptide and glucagon-like peptide-1 receptor agonist, induces substantial weight loss in patients with obesity; however, pharmacologically induced weight reduction may be accompanied by losses in fat-free mass (FFM), muscle strength (MS), and resting metabolic rate (RMR), potentially influencing metabolic health. The metabolic implications of short-term preservation of metabolically active tissue during TZP therapy remain incompletely characterized. Methods: We performed a secondary, exploratory analysis of a previously published 12-week prospective, non-randomized comparative study including 60 patients with obesity treated with TZP (n = 30 TZP+Low Energy Ketogenic Therapy [LEKT]; n = 30 TZP+Low Calorie Diet [LCD]). Body weight (BW), fat mass (FM), FFM, MS, and RMR were assessed at baseline and week 12. Glycaemic parameters included fasting glucose, insulin, hemoglobin A1c (HbA1c), and HOMA-IR. All analyses were exploratory and hypothesis-generating. Results: Both groups achieved comparable reductions in BW after 12 weeks. FM decreased in both groups, while relative preservation of FFM, MS, and RMR was observed in one dietary context. Short-term changes in HbA1c, insulin, and HOMA-IR were statistically associated with concurrent changes in FFM, MS, and RMR, whereas no consistent associations were observed with changes in total BW or FM. Baseline glycaemic values were largely within the normoglycemic range. Conclusions: In this short-term secondary exploratory analysis, preservation of metabolically active tissue during TZP therapy was associated with concurrent glycaemic profiles, whereas no consistent associations were observed with total weight loss magnitude. These findings do not imply causality and should be interpreted as hypothesis-generating, warranting confirmation in larger, randomized, long-term studies.

## 1. Introduction

Obesity is a chronic, progressive disease characterized by excess adipose tissue accumulation and increased cardiometabolic risk [1,2]. Pharmacological treatment options have expanded substantially with the development of incretin-based therapies, including tirzepatide (TZP), a dual glucose-dependent insulinotropic polypeptide (GIP) and glucagon-like peptide-1 (GLP-1) receptor agonist, which has demonstrated significant weight reduction in large randomized clinical trials and supporting meta-analyses [3,4,5,6,7,8,9]. Although the magnitude of weight loss is often the primary outcome in pharmacotherapy trials, increasing attention has been directed toward the composition of weight loss, particularly the preservation of fat-free mass (FFM) and resting metabolic rate (RMR). Reductions in FFM during weight loss may influence metabolic adaptation and glucose regulation, given the established role of skeletal muscle in insulin-mediated glucose disposal [10,11]. However, the short-term metabolic implications of differential preservation of metabolically active tissue during TZP therapy remain incompletely defined. We previously reported the effects of TZP combined with two dietary strategies over 12 weeks in a prospective, non-randomized study, demonstrating differences in body composition and functional parameters between groups [12]. The primary publication focused on body-composition and metabolic-rate outcomes. The present manuscript represents a secondary, exploratory analysis of the same cohort, aimed at examining whether short-term changes in glycaemic markers occur in parallel with concurrent changes in FFM, muscle strength (MS), and RMR during TZP therapy. Importantly, this analysis was exploratory and was not designed to infer causal relationships. Given the modest sample size, non-randomized allocation, and 12-week follow-up, all findings should be interpreted as hypothesis-generating. Within these constraints, our objective was to explore associations between preservation of metabolically active tissue and short-term glycaemic profiles during TZP-induced weight loss.

## 2. Materials and Methods

### 2.1. Study Design and Secondary Exploratory Analysis

This study represents a secondary, exploratory analysis of data derived from a previously published prospective non-randomized comparative study evaluating the effects of TZP combined with two different dietary strategies on body composition and metabolic outcomes in patients with obesity [12]. The original investigation was conducted over a short-term 12-week period and compared TZP combined with LEKT versus TZP combined with a standard low-calorie diet (LCD). The study design, patient selection criteria, dietary protocols, and primary outcomes have been described in detail elsewhere [12]. The primary publication focused on body composition and metabolic-rate outcomes. The objective of the present secondary analysis was to explore associations between short-term changes in body composition and functional parameters (FFM, MS, and RMR) and concurrent changes in glycaemic markers during TZP therapy. This analysis was not designed to establish comparative efficacy between dietary strategies or to infer causal relationships. No additional data were collected beyond those reported in the original study, and all analyses were performed on the existing dataset.

### 2.2. Study Population

The original prospective study included 60 consecutive adults with obesity (body mass index ≥30 kg/m^2^ or ≥27 kg/m^2^ with at least one weight-related comorbidity) undergoing TZP therapy. Participants were allocated to either the TZP+LEKT group (n = 30) or the TZP+LCD group (n = 30) based on feasibility and anticipated adherence to the prescribed dietary regimen, as previously described [12]. Group assignment was pragmatic and non-randomized, reflecting routine clinical decision-making rather than an experimental allocation process. Baseline demographic and clinical characteristics, including body weight, body composition parameters, MS, RMR, and glycaemic indices, were comparable between groups. However, given the modest sample size and non-randomized allocation, potential baseline imbalances cannot be entirely excluded. All participants completed the 12-week intervention period, and no dropouts were reported. For the present secondary exploratory analysis, the entire cohort from the original study was included without additional selection. Because of the non-randomized design and absence of a placebo or control arm, findings should be interpreted within the framework of an observational comparative study and do not allow causal inference.

### 2.3. Tirzepatide Treatment and Dietary Interventions

As described in the original study, TZP was administered subcutaneously once weekly, starting at a dose of 2.5 mg for the first four weeks and increased to 5 mg for the subsequent eight weeks [12]. The pharmacological treatment protocol was identical in both groups. Dietary interventions consisted of either LEKT or LCD. The LCD was characterized by an energy intake of approximately 1200 kcal/day, with a macronutrient distribution of approximately 50% carbohydrates, 20% protein, and 30% fat, in accordance with standard clinical recommendations. The LEKT protocol provided a comparable caloric intake (~1200 kcal/day) but was characterized by marked carbohydrate restriction (<30 g/day), higher protein intake (~1.3 g/kg of ideal body weight), and a higher proportion of dietary fat, with the intent of promoting nutritional ketosis. Participants were instructed to maintain their habitual physical activity levels throughout the study period [12]. Objective measures of dietary adherence or biochemical confirmation of nutritional ketosis were not systematically collected as part of the original study. The present secondary exploratory analysis did not modify the intervention protocols and relied entirely on data collected within the framework of the original investigation.

### 2.4. Assessment of Anthropometric, Body Composition, and Functional Parameters

Body weight (BW) and height were measured under standardized conditions. Body composition was assessed using multi-frequency bioelectrical impedance analysis (BIA), as previously described [12], from which fat mass (FM) and fat-free mass (FFM) were derived. BIA measurements were performed according to manufacturer recommendations under standardized hydration and fasting conditions. Resting metabolic rate (RMR) was measured by indirect calorimetry using a metabolic monitor under standardized conditions, including fasting and a thermoneutral environment. Muscle strength (MS) was evaluated using handgrip dynamometry of the dominant hand, recording the highest value obtained from repeated measurements as absolute MS. All measurements were obtained at baseline and at the end of the 12-week intervention period. This secondary analysis relied exclusively on these pre-specified measurements.

### 2.5. Assessment of Glycemic and Biochemical Parameters

Glycaemic parameters included fasting plasma glucose, fasting insulin, hemoglobin A1c (HbA1c), and the homeostasis model assessment of insulin resistance (HOMA-IR). HOMA-IR was calculated using the standard formula derived from fasting glucose and insulin concentrations. Blood samples were collected under fasting conditions at baseline and after 12 weeks and analyzed in a certified laboratory using standardized assays, as described in the original study [12]. HbA1c was measured using a standardized NGSP-certified assay in the hospital clinical laboratory. No additional biochemical markers were collected beyond those predefined in the primary investigation, and the present secondary exploratory analysis relied exclusively on these existing measurements.

### 2.6. Outcomes of the Secondary Analysis

The objective of this secondary exploratory analysis was to examine whether short-term changes in glycaemic markers during TZP therapy were associated with concurrent changes in body composition and functional parameters, including FFM, MS, and RMR. Given the exploratory nature of this investigation, no confirmatory hierarchical outcome structure was prespecified. Analyses focused on the description of changes in anthropometric, body composition, functional, and glycaemic variables over the 12-week period, as well as on exploratory associations between these parameters.

### 2.7. Statistical Analysis

Statistical analyses were performed using the same analytical framework as the original study [12]. Continuous variables are presented as mean ± standard deviation. Data distribution was assessed using the Shapiro–Wilk test. Within-group changes from baseline to 12 weeks were evaluated using paired samples t-tests for normally distributed variables. Between-group comparisons of changes were conducted using the Mann–Whitney U test, given the sample size and non-normal distribution of some variables. Exploratory correlation analyses between changes in glycaemic parameters and changes in body composition and functional outcomes were performed using Spearman’s rank correlation coefficients. Given the secondary and exploratory nature of the present analysis, no confirmatory statistical hierarchy was prespecified. No adjustment for multiple comparisons was performed, and the results should therefore be interpreted as hypothesis-generating. No multivariable models or baseline-adjusted analyses were conducted beyond those applied in the original study. Statistical significance was defined as a two-sided *p*-value < 0.05. *p*-values are reported descriptively and should not be interpreted as evidence of definitive treatment effects. No additional analyses beyond those reported in the original study were performed.

### 2.8. Ethical Considerations

The original prospective study was conducted in accordance with the ethical principles outlined in the Declaration of Helsinki and was reviewed according to institutional procedures and classified as negligible-risk research, as previously reported [12]. Written informed consent was obtained from all participants prior to study enrollment. The present work represents a secondary analysis of fully anonymized data derived from the original study and did not involve additional interventions or procedures on participants. No new data were collected for the purposes of this analysis. According to institutional policy, secondary analyses of anonymized datasets classified as negligible-risk research do not require separate ethical approval, and therefore no additional review was sought for the present exploratory investigation.

## 3. Results

### 3.1. Baseline Characteristics

At baseline, patients allocated to TZP combined with LEKT (TZP+LEKT) and those receiving TZP combined with LCD (TZP+LCD) were similar across assessed anthropometric, body composition, functional, and glycaemic variables. Body weight was 101.2 ± 16.5 kg in TZP+LEKT and 99.8 ± 17.1 kg in TZP+LCD (*p* = 0.74), and body mass index was 33.8 ± 4.2 vs. 34.1 ± 4.5 kg/m^2^ (*p* = 0.81). FM (36.5 ± 9.8 vs. 35.9 ± 10.1 kg; *p* = 0.86) and FFM (64.7 ± 11.2 vs. 63.9 ± 10.8 kg; *p* = 0.79) did not differ significantly between groups. MS (34.6 ± 8.1 vs. 33.9 ± 7.8 kg; *p* = 0.68) and RMR (1765 ± 215 vs. 1738 ± 228 kcal/day; *p* = 0.62) were also comparable. Glycaemic parameters at baseline were similar, including fasting plasma glucose (92.4 ± 11.6 vs. 90.8 ± 10.9 mg/dL; *p* = 0.59), fasting insulin (8.9 ± 4.2 vs. 8.6 ± 4.0 µIU/mL; *p* = 0.77), HbA1c (4.7 ± 1.1 vs. 4.3 ± 0.8%; *p* = 0.33), and HOMA-IR (1.83 ± 0.8 vs. 1.75 ± 1.4; *p* = 0.69). No statistically significant between-group differences were observed at baseline.

### 3.2. Magnitude of Weight Loss and Fat Mass Reduction

As shown in Table 1, after 12 weeks of treatment, both groups exhibited significant reductions in body weight. The percentage decrease in body weight was −10.2% ± 2.5 in the TZP+LEKT group and −9.8% ± 2.9 in the TZP+LCD group, with no statistically significant between-group difference (*p* = 0.665). FM was significantly reduced in both groups. The percentage reduction in FM was −13.4% ± 2.8 in the TZP+LEKT group and −10.2% ± 3.1 in the TZP+LCD group (*p* = 0.042). Total weight loss magnitude was similar between groups, whereas differences were observed in the relative distribution of tissue changes.

### 3.3. Preservation of Fat-Free Mass, Muscle Strength, and Resting Metabolic Rate

Differences between groups were observed when evaluating parameters related to metabolically active tissue (Table 1). In the TZP+LEKT group, FFM showed a non-significant change over the 12-week intervention (−0.50% ± 0.82; *p* = 0.487). In contrast, the TZP+LCD group exhibited a significant decline in FFM (−4.29% ± 1.31; *p* = 0.028), with a statistically significant between-group difference (*p* = 0.039). A similar pattern was observed for MS and RMR. MS showed a non-significant change in the TZP+LEKT group (−0.3% ± 0.9; *p* = 0.691), whereas a reduction was observed in the TZP+LCD group (−4.1% ± 1.2; *p* = 0.034), resulting in a between-group difference (*p* = 0.046). RMR did not significantly change in the TZP+LEKT group (−1.2% ± 0.9; *p* = 0.263) but declined in the TZP+LCD group (−5.3% ± 1.8; *p* < 0.001), with a significant between-group difference (*p* = 0.019). These findings describe differential short-term changes in FFM, MS, and RMR between dietary contexts during TZP therapy.

### 3.4. Glycemic Outcomes

Changes in glycaemic parameters over the 12-week period are summarized in Table 1. In the TZP+LEKT group, HbA1c decreased from 4.7% ± 1.1 at baseline to 3.7% ± 1.1 at follow-up (Δ −1.0% ± 0.9; *p* = 0.01). Baseline HbA1c values were within the normoglycemic range. HOMA-IR decreased from 1.83 ± 0.80 to 1.40 ± 1.10 (Δ −0.43 ± 0.70; *p* = 0.09). In the TZP+LCD group, HbA1c changed from 4.3% ± 0.77 to 4.0% ± 0.80 (*p* = 0.14), and HOMA-IR from 1.75 ± 1.40 to 1.55 ± 0.96 (*p* = 0.52). Fasting glucose and insulin concentrations did not change significantly over the study period in either group (TZP+LEKT: glucose 87 ± 21 to 80 ± 18 mg/dL; *p* = 0.17; insulin 8.5 ± 6.3 to 7.1 ± 5.2 μU/mL; *p* = 0.35; TZP+LCD: glucose 92 ± 17 to 87 ± 13 mg/dL; *p* = 0.21; insulin 7.7 ± 4.7 to 7.2 ± 3.1 μU/mL; *p* = 0.63), with no statistically significant between-group differences. These analyses do not establish causal relationships.

### 3.5. Exploratory Correlation Analyses

**Figure 1:**
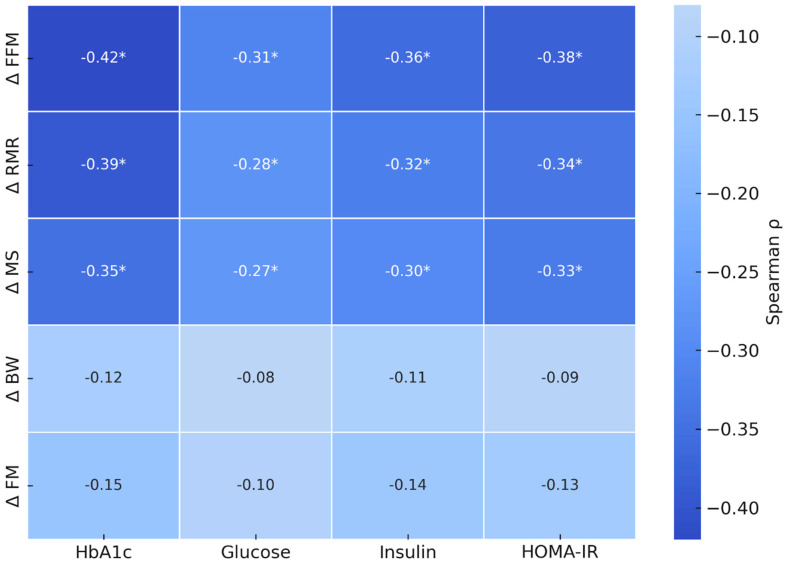
Exploratory Spearman correlation heatmap showing correlation coefficients (ρ) between changes in glycaemic markers (HbA1c, fasting glucose, insulin, and HOMA-IR) and changes in body composition and functional variables, including fat-free mass (FFM), resting metabolic rate (RMR), muscle strength (MS), total body weight (BW), and fat mass (FM). Asterisks (*) indicate statistically significant correlations (*p* < 0.05). Correlation analyses were exploratory and unadjusted. Observed inverse correlations indicate that smaller reductions in FFM, RMR, and MS were associated with lower concurrent values of selected glycaemic markers, whereas changes in total body weight and fat mass were not significantly correlated. These findings do not imply causality.

Exploratory correlation analyses were performed to examine associations between changes in body composition and functional parameters and concurrent changes in glycaemic markers across the entire cohort, irrespective of dietary assignment. Spearman correlation coefficients (ρ) and corresponding *p*-values are reported in Figure 1. Changes in HbA1c were inversely correlated with changes in FFM (ρ = −0.34; *p* = 0.009) and RMR (ρ = −0.29; *p* = 0.02), and with MS (ρ = −0.27; *p* = 0.04). Similarly, inverse correlations were observed between changes in insulin and HOMA-IR with changes in FFM (insulin: ρ = −0.30; *p* = 0.03; HOMA-IR: ρ = −0.33; *p* = 0.01) and RMR (insulin: ρ = −0.28; *p* = 0.04; HOMA-IR: ρ = −0.31; *p* = 0.02). No statistically significant correlations were observed between changes in total body weight or FM and changes in glycaemic parameters (all *p* > 0.1). These analyses were exploratory and unadjusted for potential confounders, including dietary group assignment, and therefore do not establish directionality or causality. Additional exploratory correlation analyses were performed separately within each dietary group (TZP+LEKT and TZP+LCD). As shown in Supplementary Figure S1, the overall directional patterns of the correlations were broadly consistent with those observed in the pooled analysis, although statistical significance was reduced because of the smaller sample size within each subgroup.

## 4. Discussion

Tirzepatide has demonstrated substantial weight loss in large randomized clinical trials, with approximately 70–75% of weight reduction attributable to FM and the remainder to FFM loss over longer follow-up periods [6,7,8,9]. However, the short-term metabolic correlates of differential changes in metabolically active tissue during TZP therapy remain insufficiently explored. In this secondary, exploratory 12-week analysis [12], we observed that reductions in HbA1c and indices of insulin resistance occurred alongside differential changes in fat-free mass (FFM), muscle strength (MS), and resting metabolic rate (RMR). Importantly, total weight loss magnitude was similar between dietary contexts, whereas short-term alterations in metabolically active tissue differed. These observations should be interpreted with considerable caution, given the exploratory design, modest sample size, and absence of multiplicity adjustment. This exploratory study was not designed to establish causal relationships or comparative efficacy between dietary strategies. Correlation analyses were exploratory and unadjusted for potential confounders. Given the exploratory design and modest sample size, multivariable or partial correlation analyses were not performed. Therefore, the reported associations should be interpreted as unadjusted exploratory signals rather than independent relationships. Accordingly, the findings should be regarded as hypothesis-generating. Large, randomized trials such as SURMOUNT-1 [6] have demonstrated that tirzepatide induces pronounced fat mass loss with proportional, though not negligible, reductions in FFM over 72 weeks. Compared with those datasets, the current study is substantially smaller and shorter in duration and therefore cannot inform long-term tissue adaptation or durability of glycaemic effects. Rather, our findings describe short-term associations observed within a pragmatic clinical context. An additional consideration relates to baseline metabolic status. Participants were largely normoglycemic at baseline, with HbA1c values within the reference range. Accordingly, absolute HbA1c values in this cohort were expected to fall toward the lower end of the normal distribution. The absolute reductions in HbA1c observed in one group, although statistically significant, were modest in magnitude and of uncertain clinical relevance in this population. Particularly in individuals with baseline values within the reference range, small fluctuations may reflect regression toward the mean, analytical variability, or short-term metabolic adaptation rather than clinically meaningful glycaemic improvement. These considerations further reinforce the need for cautious interpretation, particularly given the exploratory design, modest sample size, absence of multiplicity adjustment, and baseline normoglycemia. Accordingly, the present findings should not be interpreted as evidence of risk reduction, durable metabolic benefit, or superiority of one dietary approach over another. Recent mediation analyses suggest that the glycaemic effects of tirzepatide are mediated by both weight-dependent and weight-independent mechanisms [3,4]. The present exploratory findings are compatible with the possibility that body composition changes may contribute to short-term glycaemic profiles; however, they do not exclude the central role of overall weight loss. Importantly, the absence of significant correlations between total body weight change and glycaemic markers in this dataset should not be interpreted as evidence against weight-mediated mechanisms, particularly given the limited statistical power. From a physiological perspective, skeletal muscle mass and resting metabolic rate are closely linked to insulin-mediated glucose disposal [10,11]. Nevertheless, the current analysis does not provide mechanistic evidence, and any interpretation regarding muscle-driven metabolic modulation remains speculative. Several limitations warrant explicit acknowledgment. First, the study was non-randomized and observational in design, with pragmatic allocation based on anticipated adherence [12]. This allocation strategy may introduce selection bias, as participants considered more likely to adhere to the dietary intervention could have been preferentially assigned to one group. In addition, potentially relevant confounding variables, such as patient motivation, previous dietary experiences, lifestyle behaviors, or other psychosocial characteristics, were not systematically assessed. These unmeasured factors may have influenced both adherence to the dietary intervention and metabolic outcomes, and therefore could partially contribute to the associations observed in this exploratory analysis. Second, the sample size was modest (n = 60), limiting statistical power and increasing the risk of type I error. Third, no formal correction for multiple comparisons was applied. Because no formal correction for multiple comparisons was applied, the reported *p*-values should be interpreted strictly as exploratory and hypothesis-generating. These statistical signals should not be considered evidence of a confirmed statistical or clinical effect and require confirmation in larger, appropriately powered studies with prespecified analytical plans and multiplicity control. Fourth, objective biomarkers of dietary adherence or nutritional ketosis were not systematically collected. Another limitation relates to the use of bioelectrical impedance analysis (BIA) for body composition assessment. BIA estimates are influenced by body water distribution and hydration status. Both tirzepatide therapy and ketogenic dietary interventions may induce changes in fluid balance, particularly during the early phases of treatment. Consequently, shifts in hydration status may have affected impedance-derived estimates of fat-free mass, potentially influencing the observed associations between changes in body composition and metabolic markers. Fifth, the follow-up duration was limited to 12 weeks, precluding assessment of long-term persistence or clinical durability of the observed associations. Within these constraints, this short-term exploratory analysis suggests that concurrent preservation of metabolically active tissue during TZP therapy may be associated with short-term glycaemic profiles. The present analysis should therefore be interpreted as an exploratory extension of our prior report [12] rather than as an independent confirmatory investigation, thereby generating hypotheses for future investigation. Larger, randomized, and longer-term studies incorporating baseline-adjusted models, multiplicity control, and objective dietary adherence measures are required to clarify whether modulation of body composition meaningfully influences metabolic outcomes during incretin-based pharmacotherapy.

## 5. Conclusions

In this short-term secondary exploratory analysis, changes in glycaemic markers during tirzepatide therapy were observed in parallel with differential alterations in body composition and functional parameters. Total weight loss magnitude was comparable across dietary contexts, whereas short-term preservation of metabolically active tissue differed. These findings describe exploratory associations and do not establish causality or comparative efficacy between dietary strategies. The results should therefore be interpreted cautiously and considered hypothesis-generating. Further adequately powered, randomized, and longer-term studies are required to determine whether modulation of body composition meaningfully influences metabolic outcomes during incretin-based pharmacotherapy.

## Figures and Tables

**Table 1:** Baseline and 12-Week Values of Clinical Parameters. Continuous variables are presented as mean ± standard deviation (SD). *p*-values refer to within-group comparisons (baseline vs. 12 weeks). Between-group comparisons of changes are reported in the manuscript text. Given the exploratory nature of the analysis, no adjustment for multiple comparisons was performed. Baseline HbA1c values were within the normoglycemic range in both groups.

Parameter	Group	Baseline	12-Week Follow-Up	*p*-Value
Body weight (kg)	TZP+LEKT	101.2 ± 16.5	90.9 ± 15.8	<0.001
	TZP+LCD	99.8 ± 17.1	90.0 ± 16.3	<0.001
Fat mass (kg)	TZP+LEKT	36.5 ± 9.8	31.6 ± 9.2	<0.001
	TZP+LCD	35.9 ± 10.1	32.3 ± 9.7	<0.001
Fat-free mass (kg)	TZP+LEKT	64.7 ± 11.2	64.4 ± 11.0	0.487
	TZP+LCD	63.9 ± 10.8	61.1 ± 10.2	0.028
Fasting glucose (mg/dL)	TZP+LEKT	87 ± 21	80 ± 18	0.17
	TZP+LCD	92 ± 17	87 ± 13	0.21
Insulin (µIU/mL)	TZP+LEKT	8.5 ± 6.3	7.1 ± 5.2	0.35
	TZP+LCD	7.7 ± 4.7	7.2 ± 3.1	0.63
HOMA-IR	TZP+LEKT	1.83 ± 0.80	1.40 ± 1.10	0.09
	TZP+LCD	1.75 ± 1.40	1.55 ± 0.96	0.52
Hemoglobin A1c (%)	TZP+LEKT	4.7 ± 1.1	3.7 ± 1.1	0.01
	TZP+LCD	4.3 ± 0.77	4.0 ± 0.80	0.14

HOMA-IR, homeostasis model assessment of insulin resistance.

## Data Availability

The data included in this manuscript were derived from the University database. We are not authorized to share the data with third-party organizations. However, the corresponding author is available to provide any explanation to the Editor if requested.

## Supplementary Materials

The following supporting information can be downloaded at: https://www.mdpi.com/article/10.3390/nu18071092/s1. Figure S1. Stratified exploratory correlation analyses by dietary group. Exploratory scatter plots show-ing associations between changes in HbA1c and changes in fat-free mass (FFM), resting metabolic rate (RMR), and mus-cle strength (MS) analyzed separately for each dietary group. Upper panels: TZP+LEKT (n = 30). Lower panels: TZP+LCD (n = 30). Each panel reports the corresponding Spearman correlation coefficient (ρ) and p-value. Correlation analyses were exploratory and unadjusted. TZP: Tirzepatide; LCD: Low Calorie Diet; LEKT: Low Energy Ketogenic Therapy.
