# Supplementary material for: Short-Term Associations Between Fat-Free Mass Preservation and Glycaemic Markers During Tirzepatide Therapy: A Secondary Exploratory Analysis
Source: Nutrients. 2026 Mar 29;18(7):1092. doi: 10.3390/nu18071092 (PMC13074557; doi:10.3390/nu18071092)
Supplement: Supplementary file 1 [file nutrients-18-01092-s001.zip › nutrients-4201723-supplementary.pdf]

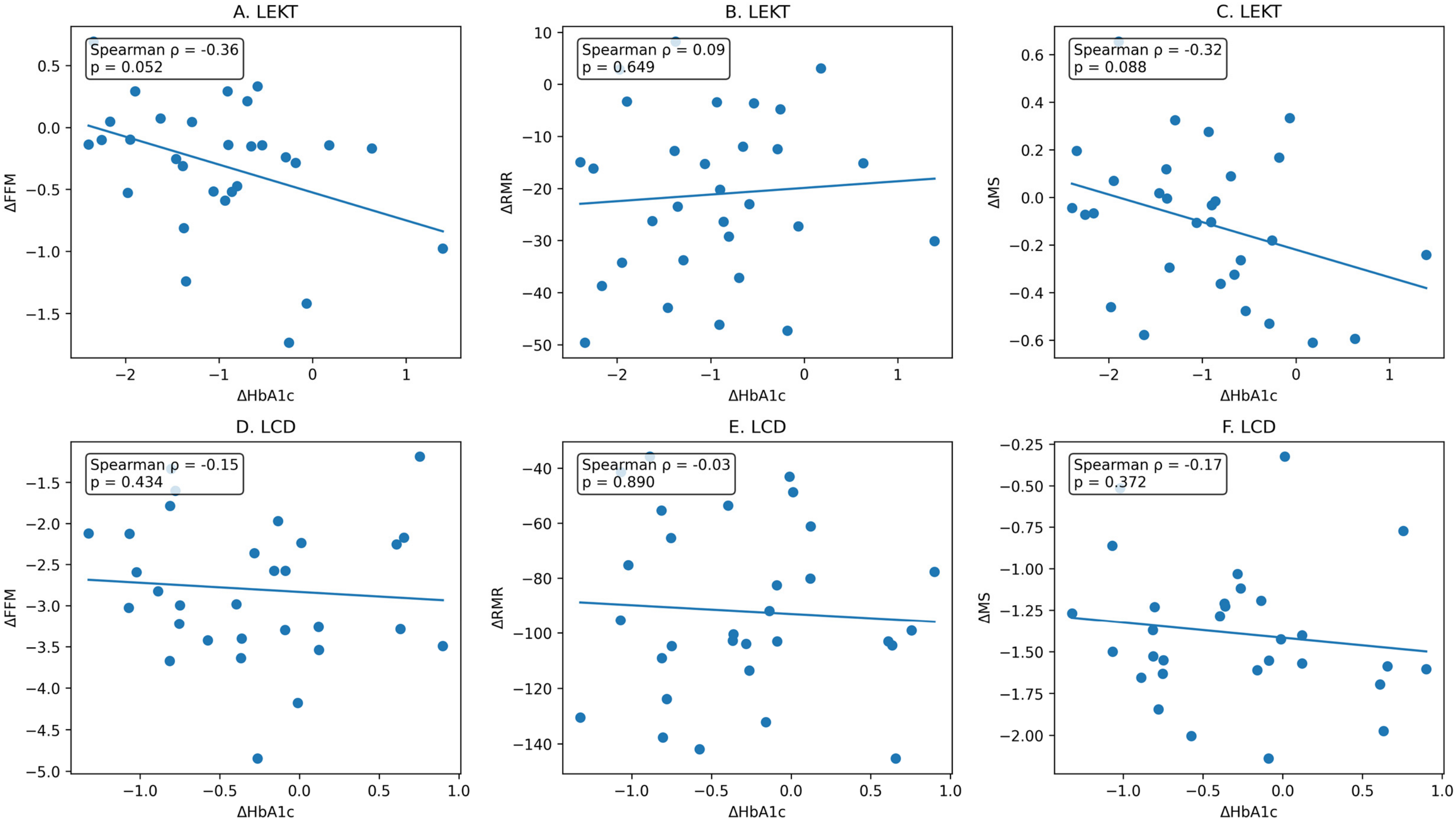

Supplementary Figure S1. Stratified exploratory correlation analyses by dietary group. Exploratory scatter plots showing associations between changes in HbA1c and changes in fat-free mass (FFM), resting metabolic rate (RMR), and muscle strength (MS) analyzed separately for each dietary group. Upper panels: TZP+LEKT (n = 30). Lower panels: TZP+LCD (n = 30). Each panel reports the corresponding Spearman correlation coefficient (ϱ) and p-value. Correlation analyses were exploratory and unadjusted. TZP: Tirzepatide; LCD: Low Calorie Diet; LEKT: Low Energy Ketogenic Therapy.
